# Analysis of allergen components and identification of bioactivity of HSP70 in pollen of *Populus deltoides*

**DOI:** 10.1186/s12953-021-00178-8

**Published:** 2021-09-03

**Authors:** Wei Guo, Xiaodong Zhan, Feng Jiang, Yilong Xi

**Affiliations:** 1 School of Ecology and Environment, Anhui Normal University, Wuhu, 241002 China; 2 Department of Parasitology, School of Basic Medicine, Wannan Medical College, Wuhu, 241002 China

**Keywords:** *Populus deltoides* pollen, Proteosome, Hsp70, Allergen, Allergic disease

## Abstract

**Background:**

Allergies caused by pollen from *Populus deltoides* are common, but the allergic components are still unclear.

**Methods:**

The total proteins in pollen of *P. deltoides* were analyzed by proteomics, and the potential allergens were identified via the WHO/IUIS database and the allergenOnline database retrieval. One target protein was screened by bioinformatics and expressed in *Escherichia coli.* The biological activity of the expressed product was verified by animal experiments.

**Results:**

The total of 3929 proteins in pollen of *P. deltoides* were identified, and 46 potential allergens belonging to 10 protein families were recognized by database retrieval. B9N9W6 protein of Hsp70 family was screened by bioinformatics analysis and expressed successfully. ELISA showed that B9N9W6 can stimulate the immune system to produce specific IgE and promote the generation of IL-4. Flow cytometry showed that B9N9W6 can significantly stimulate the proliferation of CD4^+^ T cells and promote the polarization of Th2 cells. The pathological sections of mice lung tissues indicated that alveolar destruction was more severe in the B9N9W6 group than that of extract group, and there were more inflammatory cells infiltration, mucus exudation and bleeding.

**Conclusion:**

B9N9W6 is an important antigenic substance in the pollen of *P. deltoides*. Due to the conserved structure of Hsp70 family, more attention should be paid to the possibility of sensitization when Hsp70 from any pathogenic species is administered.

**Supplementary Information:**

The online version contains supplementary material available at 10.1186/s12953-021-00178-8.

## Background

Allergic asthma is a chronic airway inflammation disease, which characterized by chest tightness, shortness of breath, and coughing after exposure to allergens [1]. The incidence of allergic asthma has been increasing in recent years [2]. At least 300 million people suffer from allergic asthma worldwide [3], which is highest among children [4, 5]. The main pathogenesis of allergic asthma is the production of specific IgE antibody, chronic airway inflammation and airway hyperresponsiveness during the immune system responds to allergens in the environment, accompanied by the imbalance of Th1/Th2 cells and other comprehensive factors [6–8].

Pollen from plants is an important source of air-borne allergens, which seriously affects the quality of life for people who is susceptible to allergies [9]. During the period of flower opening, pollen grains are released into the air to form biological aerosols; thus, individuals are inevitably exposed to pollen. *Populus deltoides* is widely cultivated in China due to its urban greening, windbreak, and sand-fixing berm. In May of each year, mature pollen of *P. deltoides* is densely suspended in the air, which causes a mass of pollen to contact people’s eyes, nostrils, mouth and skin frequently, leading to tears, sneezing, itching and other symptoms. Our recent studies demonstrated that mature pollen of *P. deltoides* extract contains antigenic substances with strong sensitization. However, allergic components in *P. deltoides* pollen remain largely unclear.

The identification and purification of pollen allergens is of great significance for pollen allergy disease, especially in diagnosis and allergen immunotherapy (AIT). In recent years, proteomic techniques have become powerful tools for comprehensive analysis of allergen, such as Par h 1 in *Parthenium hysterophorus*, and Art an 7 in *Artemisia vulgaris* were identified by this method [10, 11]. In China, proteomics was used to analyze and compare the possible allergens in mutants of *P. deltoides* [12, 13]. Wang et al. have analyzed the possible allergen components in *Populus tomentosa* by proteomics [14]. However, a systematic experimental basis is lacking for the identification of allergens in *P. deltoides* pollen.

Hsp (Heat Shock Protein) 70 has a subtle relationship with allergic diseases. Previous studies have found that Hsp70 is an important mediator to mediate allergic reactions and is capable of binding IgE antibodies in allergic patients [15]. The levels of Hsp70 are significantly elevated in patients with allergic rhinitis [16]. Interestingly, Hsp70 is widely present in plant pollen as a pan-allergen, which could be responsible for a part of the allergenic cross-reactivity between proteins from different pollens and plant food [17]. However, the biological function of Hsp70 remains largely unknown.

To screen and verify *P. deltoides* pollen allergens, we analyzed the total protein of pollen through proteomics. Then, the sequences of identified protein were compared with the confirmed allergen via relevant allergen database to identify the allergen components in pollen. After that, Hsp70 was expressed in prokaryotic expression system and explored its biological function by animal models. As far as we know, this is the first report of comprehensive allergenic proteins and Hsp70 biological function in pollen of *P. deltoides*.

## Materials and Methods

### *P. deltoides* pollen sample

The pollen samples of *P. deltoides* used in this study were collected at the Yangtze River embankment (Wuhu, China) from Apr 20 to May 20, 2018, and stored in a refrigerator at -80°C.

### Experimental animal

A total of 30 SPF female BALB/c mice (6-weeks) were purchased from Shanghai Slack Laboratory Animal Co., Ltd. (License number: 20170005030182). The animals were kept under Specific Pathogen Free (SPF) laboratory conditions in the Hangzhou Hibio Technology Co. Ltd, with the room temperature at 22-26 °C, light and darkness for 12 hours, respectively. Animals were free to eat and drink.

### Protein extraction

Protein preparation for proteomics was as follows: The pollen samples were treated with trichloroacetic acid with a final concentration of 20% at 4 °C for 2 h, centrifuged at 12000 g at 4 °C for 3 min, and the supernatant was discarded. The protein precipitate was washed three times with pre-cooled acetone and reconstituted with 8 M urea. Then, the proteins were analyzed by 12% SDS-PAGE. Protein preparation for animal experiments was as follows: Dried pollen was extracted with PBS (solid-liquid ratio of 1 g: 20 mL) at room temperature (RT) for 24 hours. The extracted materials were precipitated at RT for 8 hours. The crude extracts were filtered through 20 μm filter paper. Then, the extracts were sterilized through a 0.22 μm strainer (Millex GP) and stored at 4°C. Finally, the protein concentration was measured by the BCA kit (Sangon, C503071-0250).

### Label-Free Quantitative Proteomics analysis of *P. deltoides* pollen

Proteomics analysis was performed with reference to relevant study methods [18]. The detailed process was as follows:

The protein solution was reduced with 5 mM dithiothreitol for 30 min at 56 °C and alkylated with 11 mM iodoacetamide for 15 min at room temperature in darkness. Then, the sample was diluted with 100 mM NH_4_HCO_3_ to urea concentration less than 2 M. Trypsin was added at 1:50 trypsin-to-protein mass ratio for the first digestion overnight and 1:100 trypsin-to-protein mass ratio for a second 4 h-digestion.

The tryptic peptides were fractionated into fractions by high pH reverse-phase HPLC using Agilent ZORBAX 300Extend C18 column (5 μm particles, 4.6 mm ID, 250 mm length), and were dissolved in solvent A, directly loaded onto a home-made reversed-phase analytical column (15-cm length, 75 μm i.d.). The gradient was comprised of an increase from 7% to 25% solvent B (0.1% formic acid in 98% acetonitrile) over 40 min, 25% to 35% in 12 min and climbing to 80% in 4 min then holding at 80% for the last 4 min, all at a constant flow rate of 700 nl/min on an EASY-nLC 1000 UPLC system. Then, the peptides were subjected to NSI source followed by tandem mass spectrometry (MS/MS) in Q Exactive^TM^ Plus (Thermo) coupled online to the UPLC. The m/z scan range was 350 to 1800 for full scan, and intact peptides were detected in the Orbitrap at a resolution of 70,000. Peptides were then selected for MS/MS using NCE setting as 28 and the fragments were detected in the Orbitrap at a resolution of 17,500.

### Assigning peptide sequences and functional annotation

The results of MS/MS data were processed by Maxquant search engine (v.1.5.2.8). Tandem mass spectra were searched against “*Populus deltoides*” database concatenated with reverse decoy database. False discovery rate (FDR) was adjusted to < 1%. The obtained MS/MS data were mapped and annotated with Maxquant software (v1.5.2.8), and all the annotated peptide data were classified by R software for gene ontology classification and KEGG signaling pathway enrichment. The thresholds for the identification of total proteins and potential allergens in *P. deltoides* pollen were determined by relevant literature [19].

### Screening of potential allergens in the Hsp70 protein family

The potential allergens of the Hsp70 protein family were screened by following steps. First, we performed T/B cell epitopes prediction for potential allergens of the Hsp70 protein family by the Immune Epitope Database and Analysis Resource (IEDB) (http://www.iedb.org/). Then, Antigenicity of the HSP70 was predicted through Vaxijen v2.0 (http://www.ddgpharmfac.net/vaxijen/VaxiJen/VaxiJen.html) (Threshold 0.4). The formula, molecular weight (MW), stability and isoelectric point (PI) for B9N9W6 were predicted via ExPASy (http://web.expasy.org/protparam/), and its solubility was analyzed by ProtScale (http://web.Expasy.org/protscale/). Thirdly, we performed sequence alignment with Hsp70s through the known allergens reported in the WHO/IUIS database and AllergenOnline database, respectively. Finally, we used Swiss-model for homology modeling to screen out Hsp70 with the highest surface complementarity with IgE antibody.

### Prokaryotic expression and purification of Hsp70

Availability of adequate amounts of the pure allergens is essential to further understand their molecular structures, which is a pre-requisite for the development of more efficacious allergen immunotherapy. The amino acid sequences and coding genes of Hsp70 were retrieved from Uniprot database. The gene of Hsp70 was synthesized and cloned into prokaryotic expression vector pET-28a (+). The expression of His-tagged recombinant Hsp70 in transformant *E. coli* BL21 was induced by IPTG (final concentration = 1 mmol/L), and the expression products were analyzed by SDS-PAGE. Then, the bacteria were collected and disrupted by sonication. Hsp70 was purified by Ni-NTA protein purification kit (Sangon, C600320-0001), and the recombinant protein was eluted with elution buffer (containing 50mM Tris, 300mM NaCl and 500mM imidazole, Ph 8.0). Then, the endotoxin was removed by Endotoxin Removal Spin Columns (Thermo Fisher, NO. 88273). Purified protein was resuspended in 1×phosphate buffered solution (PBS), and analyzed by SDS-PAGE. The protein concentration was measured by the protein concentration assay kit (Sangon, C503071-0250).

### Preparation of asthmatic model

All BALB/c mice were randomly divided into 3 groups (10 mice in each group): PBS group, pollen extract group, and Hsp70 group. Mice in extract group and Hsp70 group were injected intraperitoneally with 10 μg pollen extract or Hsp70 [dissolved in 4% (W/V) Al(OH)_3_ in PBS, endotoxin-free] on 0, 7, and 14 days, respectively, to sensitize. From the 21st day, 0.5 μg/ml pollen extract or Hsp70 suspension was sprayed for excitation for 30 min/time for one week. The PBS group was replaced with PBS containing 4% (W/V) Al(OH)_3_. Mice in each group were injected intraperitoneally with 0.8 mg BrdU (dissolved in PBS) before the last challenge. After 24 hours, blood was taken from the eye socket, left at 4°C for 2 hours, and centrifuged at 4000 g for 5 minutes. The collected serum was stored at -80°C.

### Enzyme-linked immunosorbent assay (ELISA)

The levels of total IgE antibody and IL-4 in serum were measured by ELISA kits from Songon, Inc. and Jianglaibio (Shanghai, China) (Sangon, D721094 \ Jianglaibio, JL20266) according to the manufacturer’s protocol.

### Flow cytometry

The spleens of mice in each experimental group were aseptically collected, and splenic cell suspension was prepared. The proliferation of CD4^+^ T cells and the ratio of Th1/Th2 in spleen cell suspension were detected according to the reported method [20]. In brief, the Th1/Th2 ratio in the splenic cell suspension was detected by the Th1/Th2 flow cytometry kit (Lianke Biotech, No. 70-KTH201) according to the kit’s instructions. Cell suspension was incubated for one hour at RT in the dark after adding Anti-mouse CD4-FITC antibody (Invitrogen, No. 11-0042-85) and Brdu-PE antibody (Invitrogen, No. 12-5071-41), respectively, and then determined by flow cytometry.

### Histological analysis

After the mice lung tissues were embedded in paraffin, sectioned (5μm thick), and hydrated with a series of alcohol, stained with hematoxylin and eosin (H&E), and then the changes in lung tissue structure were observed with a Nikon Ni-E microscope. Images were captured with a Nikon DS-Ri2 camera.

### Statistical analysis

Data are expressed as mean ± standard deviation (SD). Data analysis was performed with SPSS16.0 statistical software package. One-way analysis of variance (ANOVA) was used for the comparison of multiple sample means, and the least significant difference (LSD) method and Tamhane’s T2 method were used for the comparison between the two samples. The statistical test level α = 0.05.

## Results

### Proteomics of *P. deltoides* pollen and its comparison with *P. tomentosa*

**Fig. 1:**
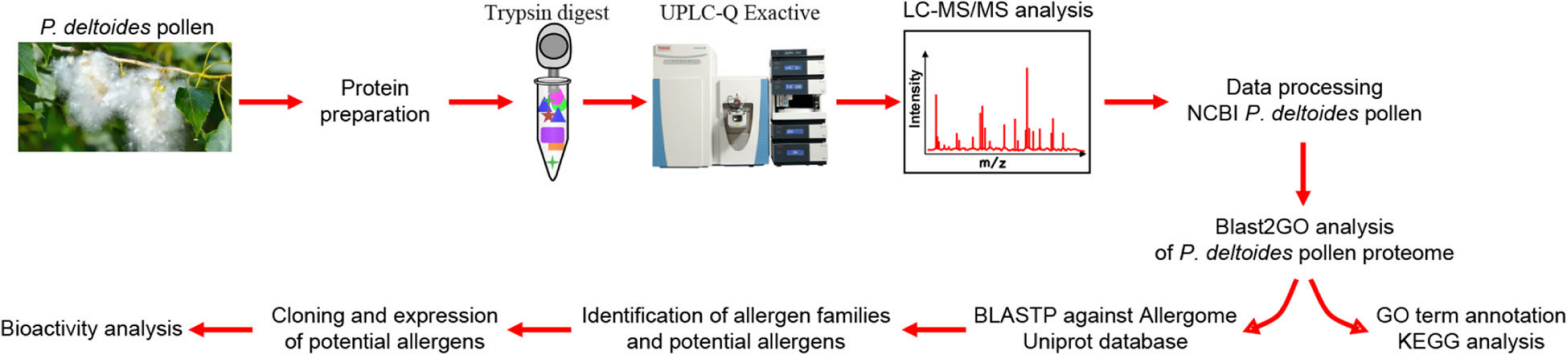
Workflow of the study to delineate the *P. deltoides* pollen proteome and analyze its bioactivity. Protein mass was mainly concentrated within 150kD, and protein sequence coverage was mainly below 40%. Peptides were mainly distributed in the length of 7-27 amino acid residues, and the peptides with length 7-17AA was relatively concentrated

In the present study, we have used workflow (Fig. 1) to classify its allergen families and to characterize new *P. deltoides* pollen allergen candidates. The GO annotation found that *P. deltoides* and *P. tomentosa* [14] have great similarities in biological process, cellular component and molecular function, but great differences in KEGG pathway (Table 1). In terms of the allergens identified, *P. deltoides* had more allergens (46 vs 27), and more members in the Hsp70 family than *P. tomentosa* (10 vs 7, Table 2).

**Table 1:** Comparison of Gene Ontology and KEGG pathways of the differentially expressed proteins (DEPs) between *P. deltoides* and *P. tomentosa*

Functional enrichment (primary)	*P. deltoides*	*P. tomentosa* [14]
Biological processes	metabolic process, cellular process and single-organism process	metabolic process, cellular process and primary metabolic process
Cellular component	cell, organelle, membrane	cell part, cell, intracellular, cytoplasm
Molecular function	catalytic activity, binding	catalytic activity, binding
KEGG pathway	ribosome, carbon metabolism, biosynthesis of amino acids	energy synthesis, protein synthesis and processing, oxidative phosphorylation, carbohydrate metabolism

**Table 2:** The information of predicted allergenic proteins in the pollen of *P. deltoides*

Allergenic protein family	Protein accession	Identity (%)	Protein description	Spectral Count(s)	Relative Abundance (%)
Pathogenesis-related proteins	A9PHF7	Hev B 6 (95.2%)	Class 4 pathogenesis-related family protein	2	0.011
Profilins	A9P8N4	Hev b 8 (94.7%)	Profilin	4	0.022
Peroxidases	B9GYJ9	Che a 3 (61.8%)	Peroxidase	10	0.05
Seed storage proteins	A0A2K1ZNE2	Gly m Bd (62.2%)	Vicilin-like seed storage protein	30	0.16
	B9H8M2	Pis v 2 (78.9%)	11S globulin seed storage protein	225	1.22
	B9HNV4	Ana o 2 (75.3%)	Vicilin-like seed storage protein	45	0.24
Proteases	A0A2K1XV49	Der p 15 (57.3%)	Protease Do-like 2 Subtilisin-like protease	2	0.011
	A0A2K2AML0	Ana c 2 (77.0%)	Cysteine protease	1	0.005
	A9PA38	Der p 15 (56.6%)	ATP-dependent Clp protease	5	0.027
	A9PBM7	CPA63 (69.4%)	Aspartyl protease Protein	28	0.152
	A9PCK2	CPA63 (68.5%)	Protein aspartic protease in guard cell	3	0.016
	B9GJE2	Per a 2 (61.1%)	Aspartic protease family protein	13	0.071
	B9GR19	CPA63 (63.5%)	Aspartyl protease family protein	6	0.033
	B9GWZ3	CPA63 (68.6%)	Aspartyl protease family protein	7	0.038
	B9H185	Pen n 13 (51.8%)	Subtilisin-like protease	6	0.033
	B9H290	Cup s 1 (57.5%)	Kunitz-type protease	13	0.071
	B9HR73	Pen n 13 (51.7%)	Subtilisin-like protease	1	0.005
	B9I8Y7	CPA63 (62.9%)	Aspartyl protease	10	0.05
	B9IKK0	Pen n 13 (56.9%)	26S protease regulatory subunit 7 family protein	14	0.076
	B9MUK8	Asp f 1 (59.6%)	26S protease regulatory subunit 7 family protein	1	0.005
	U5GXJ8	Pen n 18 (57.2%)	Subtilisin-like protease	1	0.005
Expansins	A0A2K1Z7R5	Pas n 1(68.1%)	Expansin-like A2	4	0.022
	A9PEQ7	Sor h 1(55.0%)	Expansin family protein	2	0.011
	B9H521	Lol p I (57.9%)	Alpha-expansin 11 family protein	1	0.005
	U5FK15	Cyn d 1(60.8%)	Expansin S1 family protein	1	0.005
	U5GIP5	Dac g 1(65.2%)	Expansin-like B1	1	0.005
Calcium-binding proteins	A9P988	Jun o 4 (69.6%)	Probable calcium-binding protein	7	0.038
	A9PCU6	pollen allergen group2	Calcium-binding EF hand family protein	1	0.005
Inhibitory proteins	A9P9T5	Ani s 4 (64.0%)	Cysteine proteinase inhibitor	14	0.076
	B9GNE2	Api m 6 (79.5%)	Cysteine proteinase inhibitor	2	0.011
	B9GX99	Ani s 4 (58.8%)	Cysteine proteinase inhibitor	3	0.016
	B9H290	Cry j 1(56.1%)	Kunitz-type protease inhibitor	13	0.071
	B9HSW8	Pla o 1 (63.3%)	Cell wall /vacuolar inhibitor of fructosidase	1	0.005
	B9I4L9	Ole e 3 (75.9%)	Invertase/pectin methylesterase inhibitor family protein	3	0.016
Polygalacturonase	B9GQC0	Zea m 13 (55.4%)	Polygalacturonase-like family protein	6	0.033
	B9H9W1	Cry j 2 (71.4%)	Polygalacturonase	5	0.027
Heat shock protein 70	B9GEL5	Alt a 3 (62.6%)	Heat shock protein 70	13	0.071
	B9GJ14	Der p 28 (77.4%)	Heat shock protein 70	26	0.141
	B9HMG2	Pen c 19 (90.1%)	Heat shock protein 70	11	0.060
	B9HMG7	Pen c 19 (88.7%)	Heat shock protein 70 cognate	2	0.011
	B9HMG8	Pen c 19 (91.6%)	Heat shock protein 70 cognate	9	0.049
	B9HN74	Pen c 19 (78.1%)	Heat shock protein 70	2	0.011
	B9HTJ7	Pen c 19 (90.6%)	Heat shock protein 70	1	0.005
	B9HV59	Pen c 19 (70.9%)	Heat shock protein 70	3	0.016
	B9N9W6	Der p 28 (88.2%)	Heat shock protein 70 cognate	14	0.076
	B9NBF4	Pen c 19 (91.8%)	Heat shock protein 70 cognate	9	0.049

Note: Only homologous allergen with the highest identity has been displayed

Identity, homologous allergen in the AllergenOnline database. Sum, total potential pollen allergens identified in the family

### Allergenic protein families and candidate allergenic proteins

Sequence alignment between the WHO/IUIS database and the allergenOnline database identified 46 potential allergens that belonged to 10 protein families (Table 2). The top three allergen families in relative abundance included seed storage proteins (1.63%), proteases (0.603%) and Hsp70 (0.49%). The biological activity of both seed storage proteins and proteases has been studied in-depth [21, 22], but that of the Hsp70 family is still largely unknown. It has been confirmed that Hsp70 is an important mediator to mediate allergic diseases and has the ability to specifically bind to IgE antibody [15]. However, the immunogenicity and antigenicity of Hsp70 in *P. deltoides* pollen have rarely been reported.

### Screening of potential allergens in the Hsp70 protein family

**Fig. 2:**
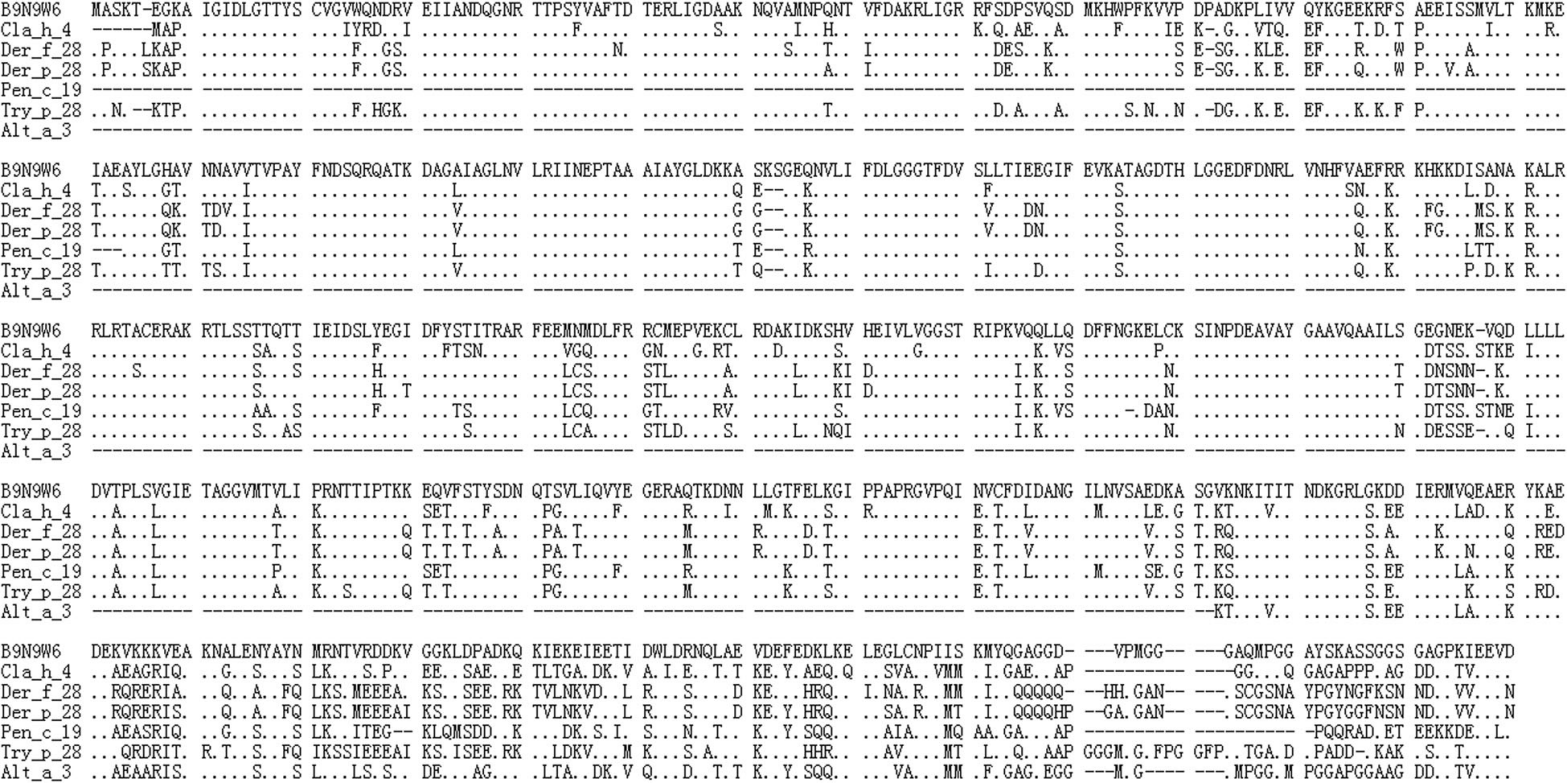
Aligning of B9N9W6 with reported allergen amino acid sequences. B9N9W6 showed a high degree of homology with the Hsp70 family of allergens. “.” represented completely identical amino acids. “-” represented no amino acids

**Fig. 3:**
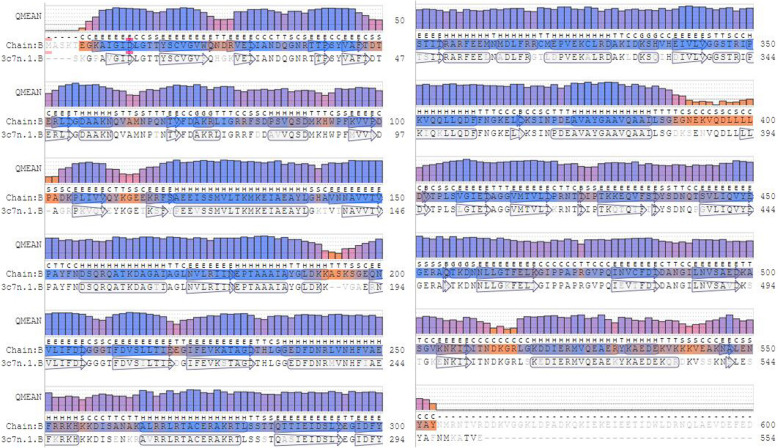
Aligning of B9N9W6 with template 3c7n.1.B amino acid sequences and secondary structure. The residues found in all protein polypeptide chains of the model are displayed in an interactive sequence display. Each residue is displayed by its one letter code below a bar chart displaying the QMEAN local quality estimation value. The secondary structure of the protein is displayed above each residue code as a single letter. B: residue in isolated β-bridge; C: loop or irregular; E: extended strand, participates in β ladder; G: 3-helix; H: α-helix; T: hydrogen bonded turn; S: bend

**Fig. 4:**
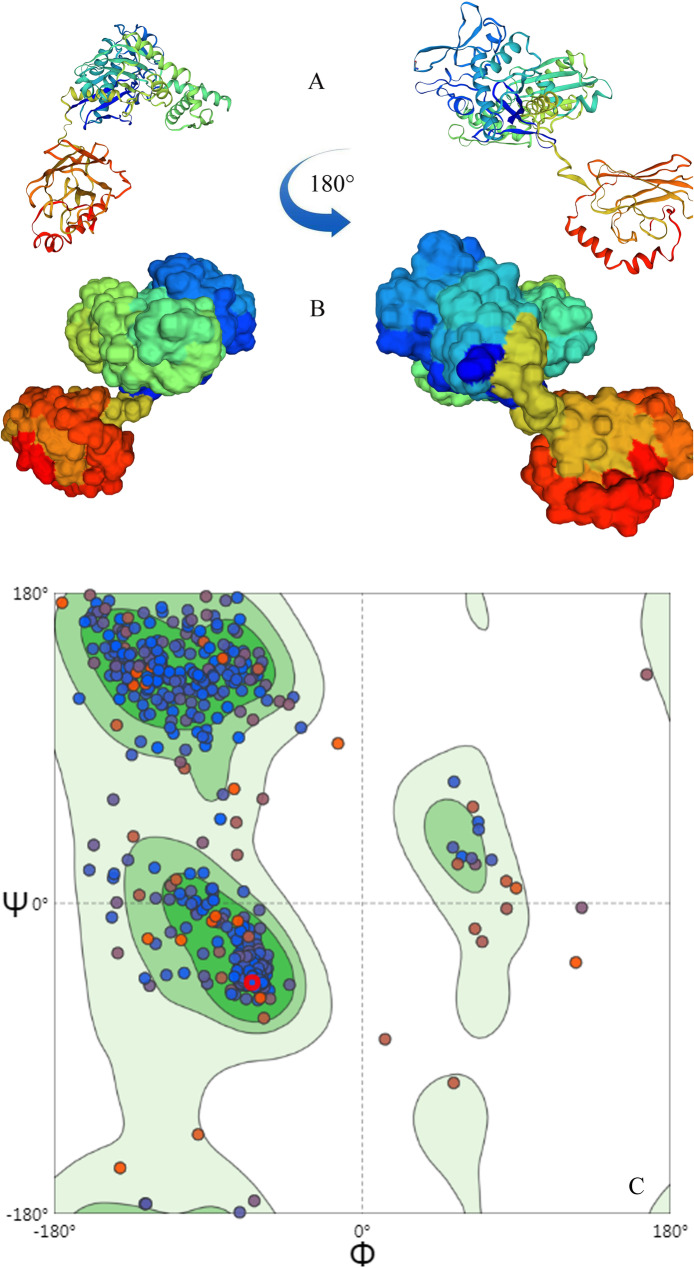
Molecular model diagram of B9N9W6 constructed by SWISS-Model. Template ID: 3c7n.1.B. QMEAN Value -0.86, GMQE Value 0.66 (The Qmean Value range is -4-0, and the closer it is to 0, the better the matching degree between the target protein and the template. The confidence range of GMQE Value is 0-1, the higher the value, the better the quality of the model). **A**: Ribbon diagram of B9N9W6. **B**: Surface structure diagram of B9N9W6. **C**: Ramachandran Plots of B9N9W6. The number of residues in favored (90.11%), in allowed regions (7.61%) and in outlier regions (2.20%), which demonstrates the high quality of the model construction

All the Hsp70s were checked, and B9N9W6 was selected as a potential allergen to elicit host immune response due to its physical and chemical characteristics as follows. IEDB epitope prediction showed that B9N9W6 had 3 T-cell epitopes and 4 B-cell epitopes with high affinity (Tables 3 and 4). The formula of B9N9W6 was C_3146_H_5062_N_880_O_1001_S_21_, MW was 71 900, PI was 5.34, and antigenicity was 0.5714 (Threshold > 0.4), which was considered as a stable hydrophilic protein. Query through AllergenOline database showed that B9N9W6 had a conserved Hsp70 domain. Protein homology analysis revealed that the Hsp70 family protein sequences were extremely conserved (Fig. 2). Secondary structure of B9N9W6 was dominated by α-helix and β ladder (Fig. 3), and it might be a hetero-dimer according to the crystal structure on the surface (Fig. 4). Compared with B9N9W6, other Hsp70 proteins are defective: B9HV59 is a hydrophobic protein, B9GEL5 is an unstable protein, B9HN74 has low immunogenicity, and B9GJ14, B9HTJ7 and B9NBF4 homologous modeling have low reliability. Although B9HMG2, B9HMG7 and B9HMG8 have high quality in terms of physical and chemical properties and immunogenicity, they have low relative abundances (Table S1). Therefore, B9N9W6 were chosen as the target protein.

**Table 3:** Prediction of T cell epitopes in B9N9W6

Epitope peptide	IC_**50**_ (SMM)	IC_**50**_ (NN)
QDLLLLDVTPLSVGI	25	21.2
LNVLRIINEPTAAAI	99	19.1
LRIINEPTAAAIAYG	176	34.5

Note: The predicted output is given in units of IC_50_nM for Stabilized Matrix Method (SMM) and Neutral-Network (NN) scoring. The lower number indicates higher affinity. Peptides with IC_50_ values < 50 nM are considered high affinity

**Table 4:** Prediction of B cell epitopes in B9N9W6

Epitope peptide	Position
GRRFSDPSVQSDMKHWPF	78-95
KASGVKNKITITNDKGRLGKDDIERMVQEAERYKAEDEKVKKKVEAKNA	499-547
NTVRDDKVGGKLDPADKQKIEKEIEETIDWLDRNQLAEVDEFEDK	557-601
QGAGGDVPMGGGAQMPGGAYSKASSGGSGAGPKI	618-651

Note: Bepipred Linear Epitope Prediction 2.0 was selected to predict the B cell Epitope, which analyzed the sequence characteristics of antigen and used the size of amino acid residues to predict the B cell epitopes

### Protein expression and purification of B9N9W6

**Fig. 5:**
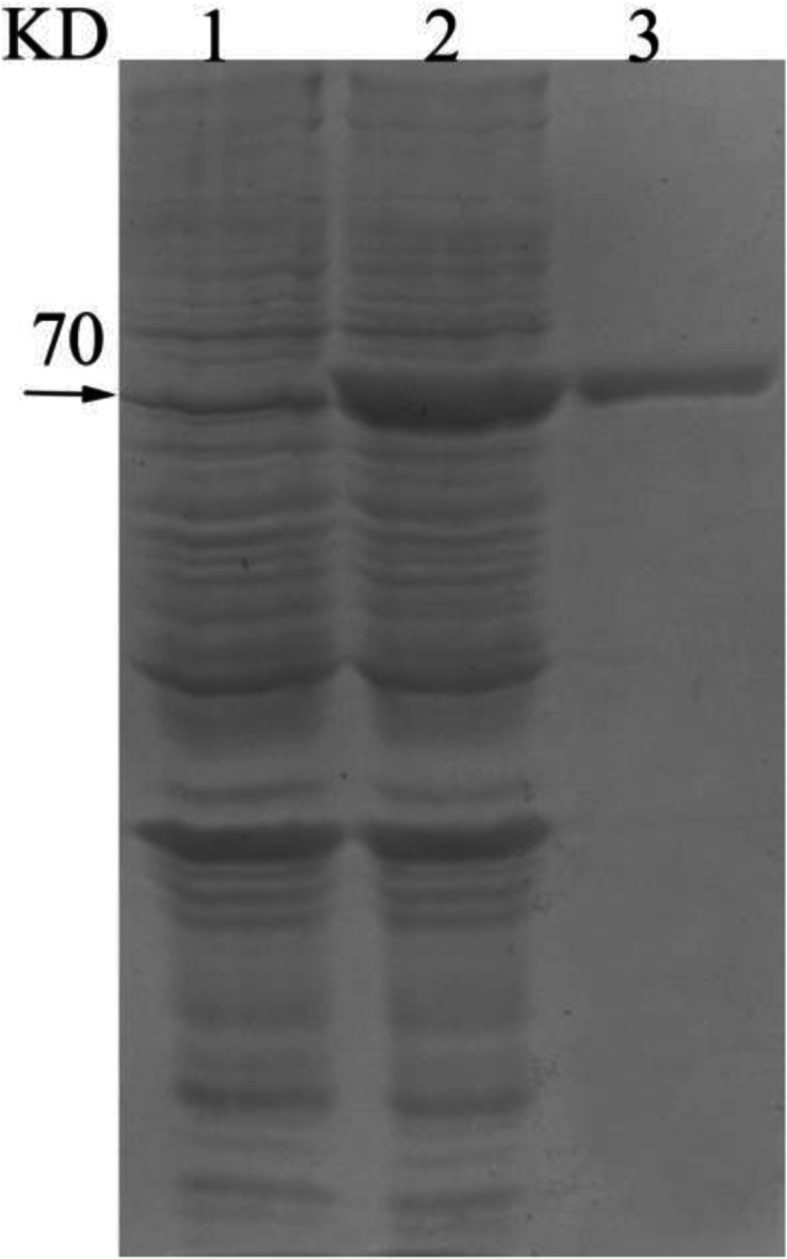
Prokaryotic expression and purification of B9N9W6. 1: *E. coli* BL21- pET-28a (+). 2: *E. coli* BL21- pET-28a- *B9N9W6*. 3: purified product of B9N9W6

SDS-PAGE of B9N9W6 showed specific band with a MW at 70KD, and purified product showed a clear band at same position (Fig. 5). The protein purity was more than 90%.

### B9N9W6 induced antibody and cytokine responses

**Fig. 6:**
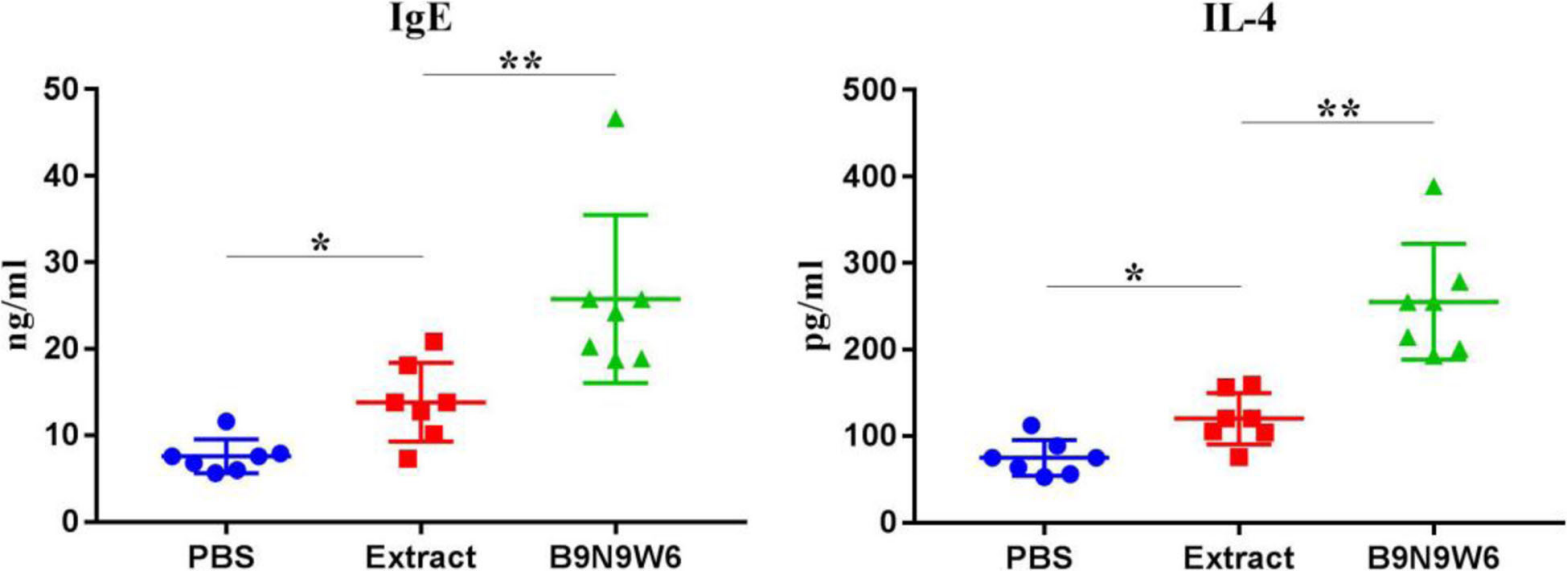
Effects of B9N9W6 on the expression of antibodies and cytokines. The vertical axis represents as the concentration of antibody or cytokine. Values are presented as the mean ± standard deviation (SD). n = 7; * *P* < 0.05; ** *P* < 0.01

In order to explore the effects of B9N9W6 on serum antibodies and cytokines, the levels of IgE and cytokine IL-4 in serum were detected by ELISA kit. The results were showed in Fig. 6. The IgE level in the extract group (13.83 ± 5.59 ng/mL) was significantly higher than that in the PBS group (7.58 ± 2.40 ng/mL, *P* < 0.05), but significantly lower than that in B9N9W6 group (25.76 ± 11.90) ng/mL, *P* < 0.01). In terms of IL-4 level, the IL-4 level in the extract group (120.08±36.58 pg/mL) was higher than that in the PBS group (74.69±25.30 pg/mL, *P* < 0.05), while the IL-4 level in the B9N9W6 group (255.24±81.88 pg/mL) was the highest among all groups (*P* < 0.01).

### B9N9W6 induced CD4^+^ T cell proliferation and Th2 differentiation

**Fig. 7:**
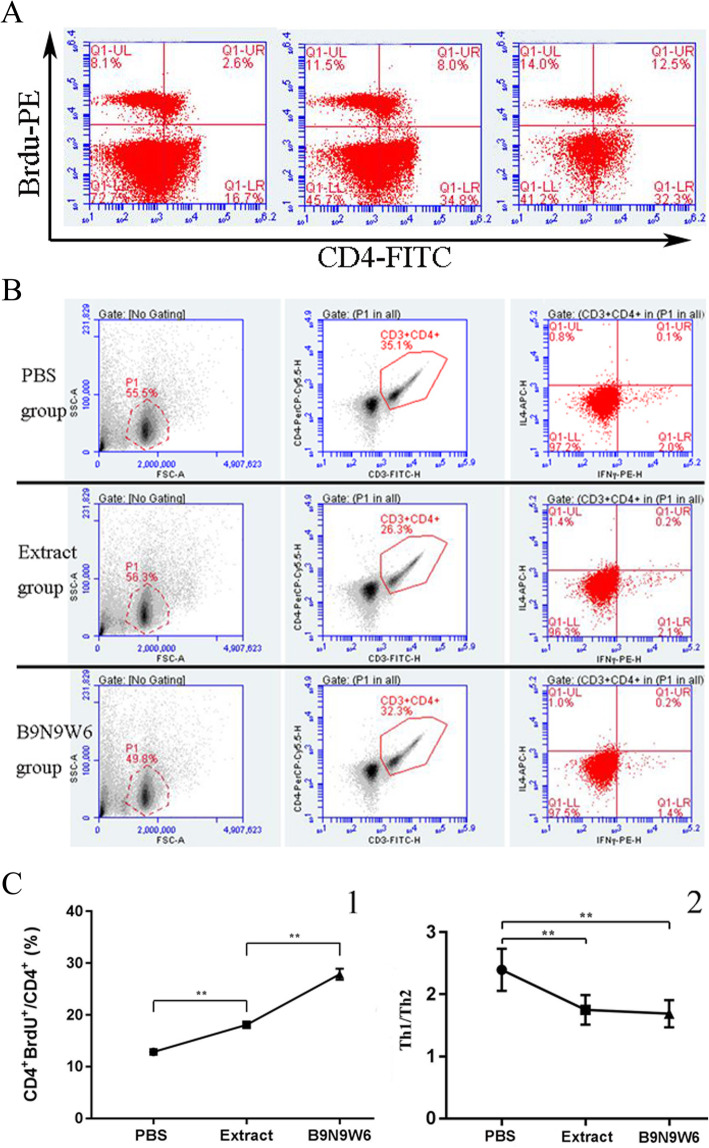
Proliferation of CD4^+^ T cells (**A**) and polarization of Th2 (**B**) both treated with B9N9W6. C1: Proportion of CD4^+^ BrdU ^+^ T cells in terms of total CD4^+^ T cells in each experimental group. C2: Ratio of Th1/Th2. Values are presented as the percentages. ** *P* < 0.01

In order to evaluate the ability of B9N9W6 to stimulate the proliferation of CD4^+^ T cells, we detected the number of proliferating CD4^+^ T cells and their subsets by flow cytometry. The results showed that compared with the PBS group (12.84 ± 0.53 %) and extract group (18.10 ± 0.58 %), B9N9W6 significantly promoted the proliferation of CD4^+^ T cells (27.86 ± 1.07 %, *P* < 0.01) (Fig. 7A, C1). Compared with the PBS group (2.39 ± 0.34), the ratio of Th1/Th2 in extract group (1.75 ± 0.24) and B9N9W6 group (1.69 ± 0.21) had significantly lower (*P* < 0.01) (Fig. 7B, C2).

### B9N9W6 induced allergic inflammation in the lung tissue of mice models

**Fig. 8:**
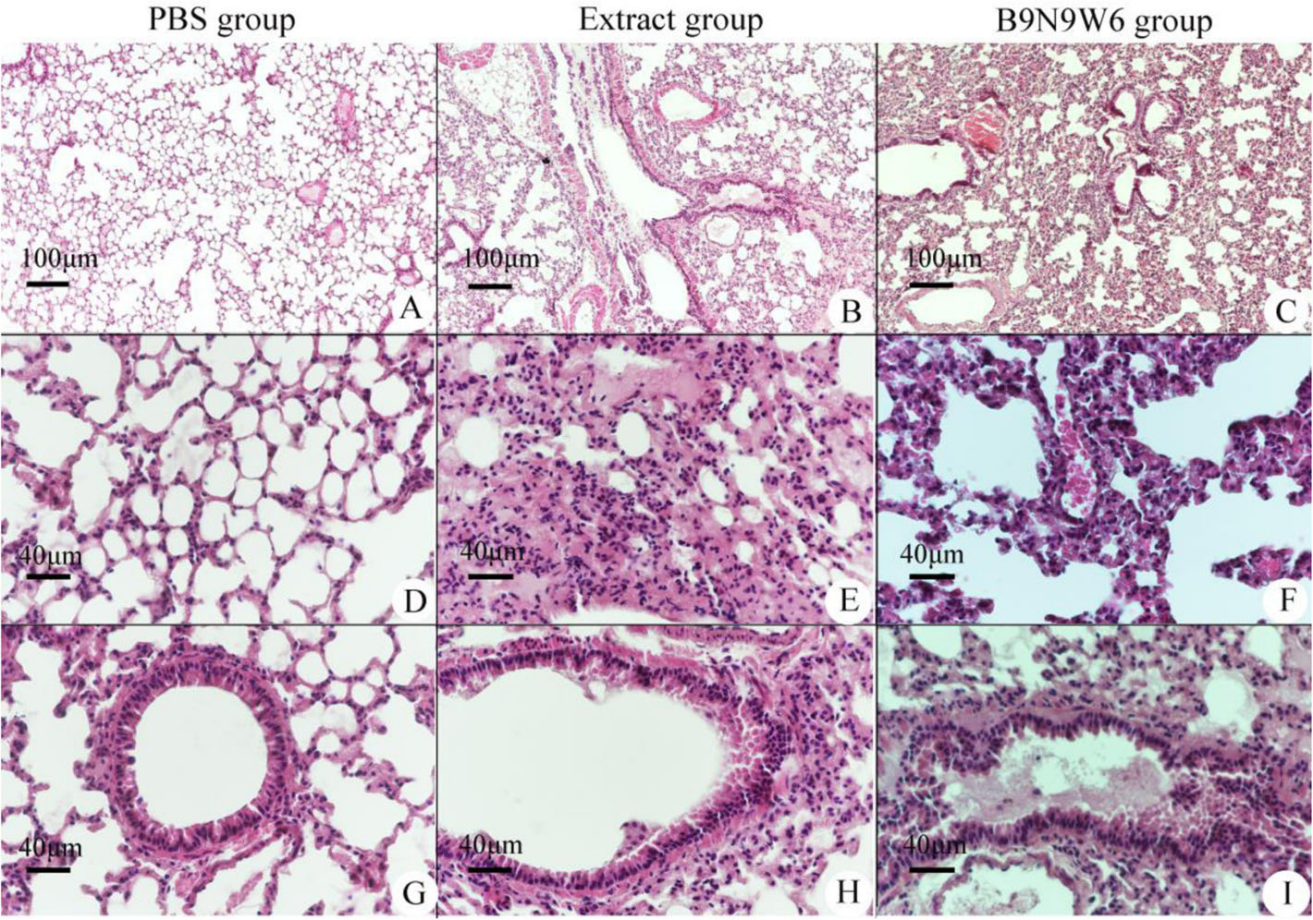
Inflammation and allergic pathological features in lung tissue of mouse model treated with B9N9W6. **A**-**C**: lung tissue (100×). **D**-**F**: alveolar (400×). **G**-**I**: terminal bronchus (400×)

Compared with the PBS group, the extract group showed the number of lung tissue alveoli decreased due to destroyed anatomical structure, and increased mucus in the respiratory bronchus (Fig. 8B). A large number of inflammatory cells were exudated to the lung field. Alveolar septum thickens due to edema while mucus and inflammatory cells appeared in the terminal bronchioles and its periphery (Fig. 8E, H). Compared with the extract group, a mass of red blood cells exudate was found in the B9N9W6 group, and the destruction of alveoli was further aggravated (Fig. 8C); Bullae formed, the collapsed alveolar cavity and exudate of erythrocyte were detected; Mucus and inflammatory cells were abundant in the terminal bronchioles (Fig. 8F, I).

## Discussion

In recent years, allergic diseases caused by pollens have attracted much attention, especially in the plant species releasing pollens, specific IgE reactivity and the influence of air pollutants on pollen transmission [23–25]. Nevertheless, identification of allergenic proteins and their bioactivity have remained elusive. In this study, we detected 3929 distinct proteins in pollen of *P. deltoides* by proteomics. We performed systematic bioinformatics analysis of all identified proteins, including protein annotation, functional classification and functional enrichment. Through functional annotation, we found that the total proteins of *P. deltoides* pollen and *P. tomentosa* pollen have great difference in function [14]. GO categorization showed that the total proteins of *P. deltoides* pollen were significantly different from those of its two mutants in biological process, molecular function, and cellular component [12]. KEGG enrichment analysis indicated that these identified proteins were not only involved in organelle composition and biogenesis, but also in biological processes such as metabolism and synthesis. There are great differences in the ingredients and functions of pollen proteins among different species of the same genus, which provides a material basis for antigen screening.

Plant-derived allergens mainly belong to disease-related protein 10 (DRP-10), thomas protein-like protein (TLP), Non-specific lipid transfer proteins (nsLTPs), expansion proteins, calcium binding proteins and profilin protein families [26, 27]. These proteins are called pan-allergen in specialized terms such as profilin, because the same family of proteins has a common antigenic determinant, and they can cause a wide range of cross-reactions [28]. Proteins of the same family share a common domain and are relatively conservative in structure, which caused the common allergen proteins that can be identified in a variety of plants [29–31]. In this study, through sequence alignment in the database, we identified 46 potential allergens belonging to 10 protein families. The top three allergen families in relative abundance are the seed storage proteins, protease family and the Hsp70 family. These results indicated that *P. deltoides* pollen contained abundant protein components. As pan-allergens, these proteins are widely found in pollen and plant fruits, and easily had fruit-vegetable-pollen cross-reactive allergy syndromes.

Hsp represent a family of molecular chaperones that respond to refolding proteins, protein trafficking, and cell signaling processes [32–34]. Hsp70 is an important member of the Hsp family involved in stress response, which often used as a potential biomarker, therapeutic target, or modulator of inflammation [35, 36]. Furthermore, Hsp70 is also involved in leaf remodeling, flowering and disease resistance in plant [37–39]. A correlation between biological function and allergenic capacity of proteins related to stress response has not been clearly demonstrated. Studies had shown that luminal binding protein of Hsp70 family in hazel pollen is a cross-reactive allergen [17]. C-terminal region of Hsp70 of *Echinococcus Granulosus* is antigenic molecule inducing both B and T cell responses [15]. Coincidentally, a large number of proteins and potential allergens containing the Hsp70 domain were identified in this study, such as B9N9W6. Epitope prediction suggested that B9N9W6 might have antigenic activity. Sequence alignment showed that B9N9W6 was highly consistent with the amino acid sequences of known allergens Cla h 4, Der f 28, etc. Homology modeling for B9N9W6 found that its 3-dimensional structure was also highly similar which consists of two main useful realms separated by a hinge region (Fig. 4A, and B); which accorded with the structural characteristics of the Hsp70 family [40]. This remarkable conservation of both surface residues and main chain conformations in the Hsp70 family plays an important role in conservation of IgE-binding epitopes, and more beneficial to promote the polarization of Th2 cells [41, 42].

Identification and purification of pollen allergens is of great significance both for the study of cross-allergic reaction and AIT. The immunoreactivity of Hsp70 had been demonstrated in previous studies [17, 41, 43]. In this study, the bioactivity of B9N9W6 was detected by animal model. First, we demonstrated that B9N9W6 can stimulate the immune system to produce high levels of IgE antibodies and promote the production of IL-4 via ELISA. Allergen specific IgE antibody is a major cause of type I allergic diseases, such as asthma [44], In the experiment, we detected specific IgE produced by B9N9W6 via Western blot, and the results suggested that B9N9W6 could produce specific IgE and had the ability to bind it (Fig. S1). IL-4 is an important proinflammatory factor secreted by Th2 cells to mediate allergic airway inflammation [45]. Meanwhile, the significant increase of IL-4 concentration in the B9N9W6 group indicated the imbalance of Th1/Th2 cells and the increase of Th2 cells. Secondly, we detected the proliferation of B9N9W6 to stimulate CD4^+^ T cells and their subgroup Th1/Th2 cells by flow cytometry. It was found that B9N9W6 could significantly stimulate CD4^+^ T cell proliferation and promote Th2 cell polarization. These results suggested an immunogenicity of B9N9W6, which were consistent with ELISA. All the results above suggest that B9N9W6 may induce allergic inflammation in the airway of the mice model. To verify our hypothesis, the presence of inflammation was observed through H&E staining sections of the mice lung tissues. More inflammatory cells infiltration and mucus exudation were observed in the lung tissue of B9N9W6 group; the alveolar rupture was the most serious, and even caused pulmonary hemorrhage. Therefore, we consider that under the same concentration, the sensitization of B9N9W6 was stronger than that of extract. There are three possible reasons for this result: 1) B9N9W6 might have a dominant T/B cell epitope, which can strongly stimulate the immune response (Tables 3 and 4). 2) As a member of the Hsp70 family, B9N9W6 could enhance the activity of antibody-presenting cells (APCs) in the process of antigen processing and presentation, which is essential for the initiation and modulation of the asthmatic immune response [46]. 3) Hsp70 is a positive regulator of airway inflammation and goblet cell hyperplasia in allergic airway inflammation [47]; the pathological findings of this study supported this view. Therefore, our findings suggested that B9N9W6 potentially induces allergen and promotes Th2 inflammatory responses.

## Conclusions

In summary, this study employed the proteomics method to screen out 46 potential allergens from 10 protein families of *P. deltoides* pollen. Furthermore, B9N9W6 belongs to Hsp70 family was confirmed to have strong allergen activity and can induce allergic asthma in animal models. Our conclusions not only enrich the theoretical study of Hsp70 family in pollen allergens, but also provide reference for the study of cross-allergic reaction and immunotherapy of allergic diseases.

## Supplementary Information

**Additional file 1.**

**Additional file 2: Table S1.** Characteristics of potential allergens in HSP70 family.

## Data Availability

The datasets used and analyzed during the current study are available from the corresponding author upon reasonable request.

## Abbreviations

Hsp: Heat shock protein
AIT: Allergen immunotherapy
DRP-10: Disease-related protein 10
TLP: Thomas protein-like protein
nsLTPs: Non-specific lipid transfer proteins
SPF: Specific pathogen free
GO: Gene Ontology
KEGG: Kyoto Encyclopedia of Genes and Genomes
