# Supplementary figures and images for: Analysis of allergen components and identification of bioactivity of HSP70 in pollen of Populus deltoides
Source: Proteome Sci. 2021 Sep 3;19:10. doi: 10.1186/s12953-021-00178-8 (PMC8417992; doi:10.1186/s12953-021-00178-8)

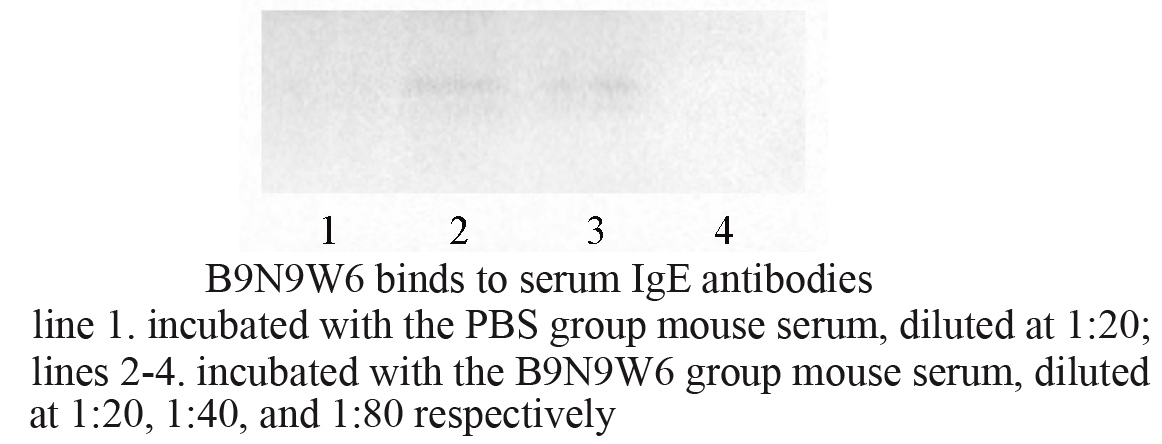

Supplement: Supplementary file 1 — Additional file 1. [file 12953_2021_178_MOESM1_ESM.jpg]
