# Supplementary material for: Analysis of allergen components and identification of bioactivity of HSP70 in pollen of Populus deltoides
Source: Proteome Sci. 2021 Sep 3;19:10. doi: 10.1186/s12953-021-00178-8 (PMC8417992; doi:10.1186/s12953-021-00178-8)
Supplement: Supplementary file 2 — Additional file 2: Table S1. Characteristics of potential allergens in HSP70 family. [file 12953_2021_178_MOESM2_ESM.docx]

**Table S1.** **Characteristics of potential allergens in HSP70 family**

|  | MW | Stability | PI | Antigenicity | Hydrophilicity | Relative abundances (%) |
| --- | --- | --- | --- | --- | --- | --- |
| B9N9W6 | 71 900 | stable | 5.34 | 0.5714 | hydrophilic | 0.076 |
| B9GEL5 | 94160 | unstable | 5.24 | 0.4785 | hydrophilic | 0.071 |
| B9GJ14 | 73209 | stable | 5.56 | 0.5707 | hydrophilic | 0.141 |
| B9HN74 | 73261 | stable | 5.56 | 0.5485 | hydrophilic | 0.011 |
| B9HV59 | 62378 | unstable | 5.31 | 0.4412 | hydrophobic | 0.016 |
| B9HMG2 | 71265 | stable | 5.14 | 0.5936 | hydrophilic | 0.060 |
| B9HMG7 | 71173 | stable | 5.09 | 0.5741 | hydrophilic | 0.011 |
| B9HMG8 | 71116 | stable | 5.13 | 0.6194 | hydrophilic | 0.049 |
| B9HTJ7 | 71131 | stable | 5.09 | 0.5777 | hydrophilic | 0.005 |
| B9NBF4 | 71139 | stable | 5.12 | 0.598 | hydrophilic | 0.049 |
